# Essential Yet Invisible: Professional Identity Formation Among Academic Internal Medicine Hospitalists

**DOI:** 10.5334/pme.2758

**Published:** 2026-06-19

**Authors:** Shazia Samanani, Beck Gold, Zareen Zaidi

**Affiliations:** 1 George Washington University School of Medicine and Health Sciences, United States

## Abstract

**Introduction:**

Academic Hospitalists (AHs) in internal medicine who are practicing in the United States (U.S.), are physicians whose inpatient clinical work is integrated with teaching, quality improvement, and research. Over 90% of US and Canada internal medicine programs rely on AHs as primary inpatient teaching attendings for medical students and residents. However, AH experience disproportionate burnout despite younger career stage, suggesting that structural and identity-related factors beyond typical career progression may drive dissatisfaction. This study explores how AHs construct their professional identity and how they view their role in the academic enterprise.

**Methods:**

Using identity theory as a conceptual framework this constructivist qualitative study used semi-structured interviews with 20 academic hospitalists across nine U.S. academic institutions during 2024–2025, selected using information power sampling to ensure sufficient data depth. Interviews explored professional roles, identity within those roles, and identity formation across distinct domains. Transcripts were coded and analyzed iteratively using Braun and Clarke’s thematic analysis.

**Results:**

Four interconnected themes were identified reflecting identity ambiguity, role strain, developmental needs, and recognition gaps among academic hospitalists: (1) the absence of consensus: defining what it means to be an Academic Hospitalist; (2) identity non-verification: the mismatch between self-perception and external recognition; (3) competing responsibilities, tensions, and burnout; and (4) mentorship and professional growth.

**Conclusion:**

AHs construct identity amid structural ambiguity and institutional non-recognition of salient professional roles. Institutions should clarify role expectations, provide non-negotiable protected time for academic engagement, establish robust mentorship infrastructure, and formally recognize educational contributions through performance metrics and compensation structures. These systemic changes are essential for supporting professional identity formation, enhancing career satisfaction, improving retention, and strengthening academic medicine’s educational mission.

## Introduction

Academic hospitalists (AHs) are inpatient internal medicine faculty who integrate clinical care with elements such as teaching, quality improvement, or research—occupy an increasingly central role in academic medical centers. This study focuses on AHs who practice within Internal Medicine. Over 90% of internal medicine departments in the US and Canada report hospitalists serving as teaching attendings for medical students and Internal Medicine residents, with many programs having the majority of inpatient teaching delivered by hospitalists [1,2,3]. Typically, AH are organized as a division or section within the Department of Medicine, though some institutions have established stand-alone Departments of Hospital Medicine [4,5]. As central contributors to both undergraduate (UME) and graduate medical education (GME), AHs deliver substantial teaching, supervision, and assessment of learners across inpatient settings.

Despite their essential contributions to learner education and patient care, AHs experience burnout and undervaluation that is disproportionate to their career stage. Recent data indicate burnout prevalence among hospitalists has risen from 13% to nearly 30% in the past 25 years, approaching rates in high-stress specialties like emergency medicine [6]. In a recent report, the American Medical Association noted hospital medicine is one of the specialties with the highest percentage of burnout [7]. AHs remain younger on average (mean age 44 as of 2012) than colleagues in other specialties, with some sources suggesting that this is due to hospitalist medicine being a newer specialty relative to others [8,9]. This paradox – high burnout among a demographically younger cohort – suggests that factors beyond career stage may be contributing to dissatisfaction and attrition in the field.

While literature examining professional identity formation (PIF) exists for medical students and early-career residents, research exploring how practicing physicians, such as AH construct professional identity remains limited [10,11,12,13]. Prior work using Bourdieusian analysis of capital, habitus, and field among medical educators in Australian and New Zealand medical schools has described educational practice as essential yet often unseen, with teaching carrying less valued forms of capital than clinical or research work. Overall, this literature is largely focused on educators within medical schools, rather than inpatient AHs whose educational contributions may be more embedded within clinical service environments. Recent research to understand the specific factors that shape professional identity among AHs has explored professional identity formation through a developmental lens, characterizing how hospitalists negotiate intrinsic and extrinsic factors, maintain a core clinician identity, and grapple with ambiguity around what it means to be “academic [14].” Further institutional reward structures have been shown to misalign with hospitalists’ professional identities, contributing to feelings of undervaluation and academic stagnation [15]. However, there continues to be a gap in the literature regarding how AHs construct and sustain professional identity amid institutional ambiguity, resource constraints, and misalignment between role expectations and identity meaning. Without this understanding, institutions cannot effectively support AH development, foster engagement, or design interventions to address burnout and retention. AHs represent a particularly salient group in which to examine PIF, given their positioning at the intersection of intensive clinical service, core educational responsibility, and academic evaluation systems that variably recognize these contributions.

We used identity theory as a conceptual framework exploring our specific research aims: (1) to identify the factors and experiences that influence professional identity formation among AHs, and (2) to understand how AHs view their role in the academic enterprise and whether this aligns with their institutional academic missions.

## Methods

### Study Design

This qualitative study employed thematic analysis and was grounded in a constructivist paradigm, which frames identity as continuously produced through individuals’ interpretations of and interactions with their social environment [16]. This stance was well suited to our aim of understanding how AHs construct professional identity within the academic enterprise, complementing Burke’s emphasis on identity as an iterative meaning-making process [17]. This approach enabled us to examine not only how AHs construct their identities but also the institutional and structural factors that enable or constrain this process. To enhance geographical and institutional diversity, we purposefully recruited participants across multiple settings in the U.S., and utilized subsequent snowball sampling to include AH from different institutions, and roles within academic medicine.

Identity formation is a topic that is complex and has many theoretical traditions [18]. Social identity theory suggests that professional identity is constructed through the roles individuals occupy and the interactions through which those roles are negotiated and validated [19]. We selected Burke’s Identity Theory because it conceptualizes identity as an ongoing feedback process between individuals and social structures, offering an analytic lens on a group like AHs whose professional identity is still being negotiated and understood within academic medicine [15]. The theory distinguishes roles – the externally defined expectations such as clinical service, teaching, and scholarly productivity from identity, the internalized meanings and standards individuals hold about who they are and what their work means. For AHs, who occupy multiple roles that are often incompletely recognized by traditional academic reward structures, this distinction allows us to move beyond describing role strain to examining how identity itself is formed, contested, and stabilized through iterative interactions with institutional feedback. In the context of AHs, this means that identity formation is dynamic, relational, and shaped by institutional structures making it both a site of vulnerability and an opportunity for meaningful institutional intervention.

### Educational Setting and Participant Sampling

The study was conducted across nine academic medical centers in the United States in 2024–2025. AHs were recruited through the author group’s specialty and professional networks using snowball sampling to ensure geographic and institutional diversity. Sample size was determined using information power, which refers to the degree to which a sample provides information pertinent to the specific study aims [20]. Given our focus on identity formation in a relatively homogenous professional group, we estimated that 20 interviews would provide sufficient information power to identify patterns while maintaining data quality and depth. This estimation was informed by preliminary literature review and refined through iterative team discussion.

Institutional Review Board approval for human subjects research was obtained from The George Washington University prior to study commencement and received an exempt status (NCR245647).

### Participant Characteristics

Twenty AHs participated; demographic characteristics are summarized in Table 1. The cohort was predominantly aged 31–40 years (70%), with 0–10 years of experience as attending physicians (85%). Most were employed in urban institutions (85%), with geographic representation from the Northeast (55%), South (25%), West (15%), and Midwest (10%). The majority held assistant professor rank (70%); 65% had engaged with institutional faculty development, 25% had completed longitudinal teaching courses, and 10% held a master’s in medical education. The cohort was 65% White, 25% Asian, 5% Black, and 5% Hispanic/Latino; 55% identified as female. Nearly all identified as straight/heterosexual (95%), and 5% were international medical graduates. Twenty-five percent had prior non-academic medicine experience; and 35% worked night shifts.

**Table 1:** Demographics table.


CHARACTERISTIC	^1^N = 20

**Age**	

<30	1 (5)

31–40	14 (70)

41–50	4 (20)

51–60	1 (5)

**Years as an Attending**	

0–5	9 (45)

6–10	8 (40)

11–15	0 (0)

16–20	2 (10)

>20	1 (5)

**Years as an Academic Hospitalist**	

0–5	11 (55)

6–10	6 (30)

11–15	0 (0)

16–20	2 (10)

>20	1 (5)

**Institution Setting**	

Rural	1 (5)

Urban	17 (85)

Blank	2 (10)

**Geographic Location**	

Northeast	11 (55)

West	3 (15)

Central/Midwest	2 (10)

South	4 (25)

**Academic Rank**	

Instructor	2 (10)

Assistant Professor	14 (70)

Associate Professor	2 (10)

Professor	2 (10)

**Level of Training in Medical Education**	

Division level training (e.g. Structured course in peer-teaching or assessment)	11 (55)

Institutional faculty development offered by your department or office of faculty affairs/deans office	13 (65)

Regional/national faculty development at national meetings	6 (30)

Longitudinal/certificate course in teaching skills	5 (25)

Master’s in education/health professions education	2 (10)

PhD in medical education	0 (0)

**Previous Work in Non-Academic Medicine**	

Yes	8 (40)

No	12 (60)

**Years Worked in Non-Academic Medicine**	

0–1	3 (38)

2–5	4 (50)

>5	1 (12)

**Night Shift Work**	

Yes*	7 (35)

No	13 (65)

**International Medical Graduate**	

Yes	1 (5)

No	19 (95)

**Race** ^+^	

Asian	5 (25)

Black/African American	1 (5)

Hispanic/Latino	1 (5)

White/Caucasian	13 (65)

Gender Identity** ^+^	

Female	11 (55)

Male	9 (45)

Cisgender	1 (5)

**Sexual Orientation** ^+^	

Straight/heterosexual	19 (95)

Gay	1 (5)


^1^n (%). *Mixed night and day shifts. **Participants were able to select >1 item, therefore values do not equal 20. ^+^Items with zero participant selections were excluded from Table 1.

### Data Collection

A semi-structured interview guide (Supplementary Material 1) was developed by the research team and grounded in Burke’s identity theory. Drawing on the theory’s distinction between externally defined roles and internalized identity standards, questions explored the roles participants occupied as AHs, the meanings they ascribed to each, and how they experienced congruence or dissonance between institutional expectations and their own identity standards.

The interview guide was pilot tested on two AHs and modified based on participant feedback. For example, pilot feedback included that one probe was leading for participants, and this particular probe was removed. All interviews were conducted virtually on Zoom by a trained research assistant (BG) and lasted approximately 45–60 minutes. Demographic information was collected prior to interviews. After obtaining informed consent, interviews were audio-recorded and transcribed using a secure transcription service. Transcripts were de-identified and reviewed for accuracy (SS and BG) before analysis. Data collection and analysis occurred iteratively, with early interviews informing subsequent interviewing and ongoing refinement of analytic focus.

### Data Analysis

De-identified transcripts were uploaded into Dedoose® a secure online qualitative analysis platform. Initial coding was informed by identity theory as a sensitizing framework, guiding analytic attention to roles, recognition, and identity negotiation, while remaining open to inductively generated codes. Braun and Clarke’s six-phase thematic analysis method was used to identify patterns in the data [21]. We performed open coding of initial transcripts, followed by consolidation of codes into a codebook. Each transcript was independently coded by two research team members (SS and BG) and coding discrepancies were resolved through discussion during team meetings. Codes were then iteratively compared across transcripts, merged or refined based on conceptual overlap, and consolidated through collaborative team discussion into themes representing coherent and distinct patterns of shared meaning.

We monitored the adequacy of our data throughout concurrent data collection and analysis using the concept of information power [20]. Several features of our study contributed to the richness of our dataset and informed our judgment that the sample held sufficient information power: a narrowly defined aim, participants with characteristics highly specific to the research question, and an analysis grounded in Identity Theory. As interviews progressed, the research team met regularly to review transcripts and discuss emerging themes. We assessed adequacy by monitoring the extent to which successive interviews generated new conceptual insights relevant to the study aims, as opposed to elaborating or confirming themes already identified. After 20 interviews, the team reached consensus that additional interviews were yielding primarily confirmatory rather than novel information, and that the dataset enabled a sufficiently rich and nuanced analysis to address the research questions. At that point, recruitment was concluded.

### Critical Reflexivity

The research team engaged in ongoing reflexive practice throughout the study, attending to how our differing positionalities shaped question design, data collection, and interpretation. SS, a woman of color and practicing AH, brought insider knowledge of hospital medicine and the academic expectations placed on AHs, which informed interview guide development and facilitated rapport during recruitment. This proximity required deliberate reflexivity to guard against assumptions of shared meaning and over-identification with participants’ experiences. ZZ, a first-generation immigrant woman of color and academic internist with inpatient and outpatient experience and expertise in qualitative research, contributed a contextually informed yet analytically distanced perspective; she frequently challenged early interpretations and pressed the team to interrogate how identity, power, and institutional structure shaped participants’ narratives. BG, a white medical student with qualitative research experience focused on health disparities and the experiences of sexual and gender minority patients, brought an outsider perspective to academic hospital medicine and conducted all interviews to reduce power differentials and encourage candid discussion. Reflexivity was operationalized through regular team meetings and written exchanges in which we examined our backgrounds, assumptions, and disciplinary commitments, and considered how these might shape our engagement with the data.

## Results

### Overview of Themes

**Figure 1:**
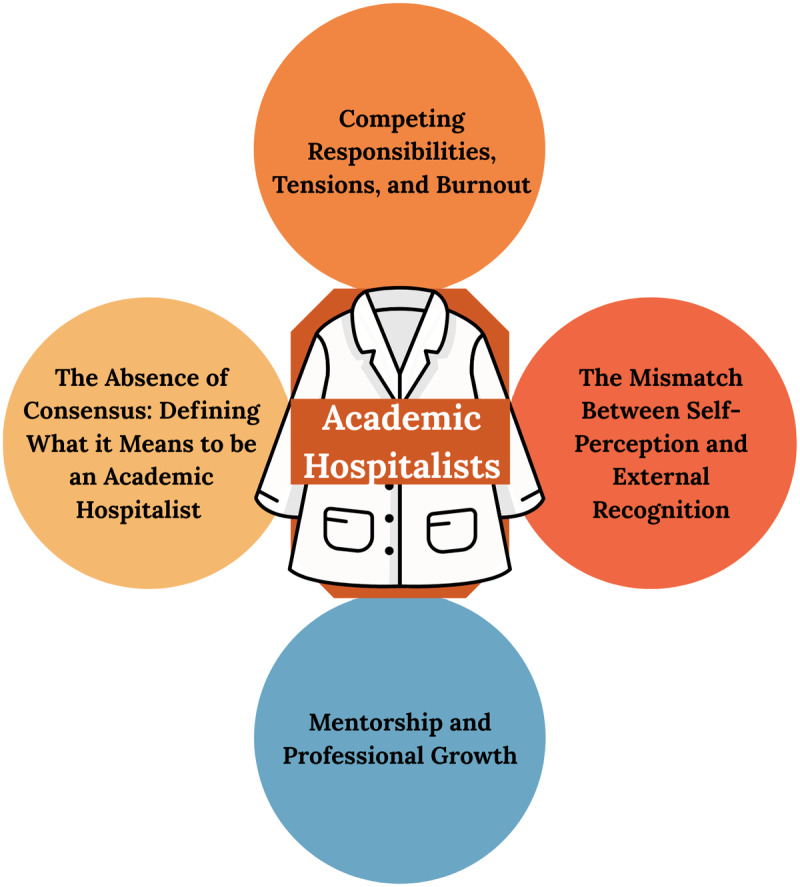
Interconnected themes illuminating the complexity of identity formation among academic hospitalists.

This study explored how AHs construct and stabilize professional identity within the evolving landscape of academic hospital medicine. Guided by Burke’s Identity Theory, the findings are organized to reflect how participants navigated the iterative feedback between their internalized identity standards and the institutional environments within which those standards were affirmed, challenged, or left unverified. The themes are therefore presented not as discrete topics but as interconnected identity processes, each illustrating how role expectations, institutional feedback, and self-perception interact to shape professional identity under conditions of ambiguity, competing demands, and inconsistent recognition. Four interrelated themes were identified (Figure 1): (1) the absence of consensus: defining what it means to be an Academic Hospitalist; (2) identity non-verification: the mismatch between self-perception and external recognition; (3) competing responsibilities, tensions, and burnout; and (4) mentorship and professional growth.

We interpret these themes as connected through a process rather than as discrete findings. The absence of a shared definition of the AH role (Theme 1) is not merely a conceptual gap; it removes the stable anchor against which AHs and their institutions might align expectations. Without that anchor, the discrepancy AHs described between how they understood their own contributions and how those contributions were recognized by others (Theme 2) becomes difficult to resolve, because there is no agreed standard to appeal to. This unresolved discrepancy was, in our reading, a precondition for the role strain participants articulated (Theme 3): competing clinical, educational, and scholarly demands are more depleting when the work itself is not legibly valued, and when AHs must continually justify a role whose boundaries are contested. Mentorship (Theme 4) functioned in participants’ accounts as the primary site where these tensions were named, interpreted, and partially absorbed — less a solution than a relational mechanism through which AHs made the ambiguity workable. Read together, the themes describe how definitional ambiguity propagates into recognition, strain, and adaptive response, which is why we present them as interconnected rather than parallel.

### Theme 1: The Absence of Consensus: Defining What it Means to be an Academic Hospitalist

AHs described pervasive definitional ambiguity about what constitutes their role, which emerged as a foundational source of identity uncertainty shaping how they understood what it means to “be” an AH and what forms of work confer academic legitimacy. While some role ambiguity exists across academic medicine, participants’ accounts pointed to a distinctive form of uncertainty rooted in the structural features of hospital medicine itself. Participants pointed out that unlike established specialties, which developed over decades alongside defined fellowship pathways, recognized scholarly traditions, and stable promotion norms, hospital medicine is a comparatively young, generalist field whose academic roles emerged in response to workforce shortages and clinical service demands rather than a coherent scholarly mandate. Without a founding disciplinary identity organized around a specific organ system, procedural domain, or research tradition, AHs have inherited academic roles whose expectations for scholarship, teaching, and productivity remain inconsistently defined across institutions and, often, within the same division. This structural ambiguity surfaced along three interrelated dimensions: whether teaching alone qualifies as academic work, whether scholarship is required, and whether academic identity is conferred by institutional location or by the nature of the work itself.

#### Ongoing Confusion and Lack of Consensus

Participants expressed uncertainty about the criteria for being considered an AH. One asked: “What is an AH is somewhat of an existential question that I think people are grappling with… is being a hospitalist and a teacher the same as being an academic hospitalist, or what is that academic side of the job?” (030). Clinical teaching was often cited as a baseline — “You can’t be an AH without teaching either on wards, on rounds, or in a more formalized setting” (007) but participants differed on whether scholarship, leadership, or teaching beyond clinical settings were also required. One distinguished between “teaching hospitalists” and “research hospitalists” (064), identifying as academic based on research engagement with minimal formal teaching. Another questioned: “In order to be an AH do you have to do research? Do you have to be on a teaching service? Or can you be an AH in terms of the fact that you do scholarly work, but your clinical time be working on your own or with PAs or NPs?” (030). Still, most agreed that “the most important thing that academic medicine, hospital medicine means to me versus being a community hospital medicine is the training and education of residents and medical students” (066).

#### Academic by Role vs. Academic by Location

Several participants challenged the assumption that institutional affiliation alone confers academic identity. One noted: “I always felt like the word academic was really just because we were at a medical school. It wasn’t because we were really doing anything, like truly academic” (016). Another described being recruited as an AH for what proved to be a “community hospitalist at an academic center” (065) with no teaching or scholarship expectations, yet still identified as an AH. This disconnect between institutional labels and lived experience had downstream consequences, particularly during transitions where expectations for “academic” work varied widely.

#### Implications for Career Mobility

The inconsistent definition of AH roles created barriers to career mobility. Without shared frameworks, prior academic contributions particularly teaching were often undervalued during transitions, requiring individuals to “reinvent the wheel.” One experienced AH described: “I come from a place where I was 100% on teaching service with residents. I was an associate program director for the residency program. I directed the medical education division… But for some reason, in my new position, I’m basically starting from scratch… I’m perceived… like any person who just graduated residency” (063). Another senior participant echoed this: “it is hard when you change institutions to get some of that guaranteed time back because you almost have to start over and reprove yourself and that can be a little bit frustrating” (018).

#### Recognizing the Inherent Educator Role of Academic Hospitalists

Despite this lack of shared understanding, participants consistently emphasized that education is core to their role, spanning informal and formal clinical teaching, didactics, and leadership positions in UME and GME. For many, educating was central to professional identity: “the way I’ve described my time is more that I was a clinician educator” (016). Others described the educator identity as emerging organically from the nature of hospital medicine: “I describe this to my residents often, like the hospitalist is the person who’s the quarterback… who has to see the field… who knows who’s on our bench and be able to work collaboratively and connected with each other. And that for me is what the educator role is, is how I can help others learn how we all can work together in a way that’s cohesive and not divisive” (064).

### Theme 2: Identity Non-verification: The Mismatch Between Self-Perception and External Recognition

AHs described a persistent mismatch between how they understood their contributions and how those contributions were recognized by institutions, which participants experienced as failed identity verification that undermined their sense of professional legitimacy.

#### Integral but Invisible

Participants viewed AHs as essential to inpatient education and patient care yet felt systematically overlooked. One captured the paradox: “We’re not appreciated. We’re not valued. We’re like second class citizens… I think that without us the hospital wouldn’t run… without us there would be no resident residency period… without us there would be a tremendous gap in student education…. So it’s this weird dichotomy where I feel like we’re underappreciated, but critical” (005). Another noted that AHs provide “probably the most amount of face time that residents and students get with really any attending…in any specialty” (018) – yet this proximity was rarely acknowledged systemically.

#### The Problem of Non-Billable Work

Teaching and mentoring, central to AH identity, were difficult to quantify and excluded from revenue-based systems. “Informal teaching is such a strong identity of AH… but you can’t quantify” (031). In RVU-driven environments, educational labor was systematically devalued: “A lot of the value that hospitalists bring… is not accounted for properly by traditional, frankly, RVU-based metrics… Educating the next generation of physicians simply does not show up in dollars until 5, 10 years down the line” (067).

#### Dismissed by Specialists and Leadership

Several participants described professional dismissal from peers and leaders. Comments such as “What would you know? You’re just a hospitalist” (064) and “You’re not as smart or as good because you’re just an internist” (016) reflected entrenched hierarchies. From leadership, participants reported lack of acknowledgment: “Leadership plays a very crucial part in terms of setting up the standards or lowering the expectations… they never measure how much is being taught” (069). In contrast, one described institutional support as transformative: “There’s such a value for hospitalists in the institution that I practice at… that makes me view myself as like an important part of the process” (054).

#### Compensation and Pay Equity

Compensation structures further undervalued generalist and educational roles. “Teaching really isn’t compensated appropriately… if it were, I think it will motivate people to do it more” (066). Another argued that “academic hospitalists are the backbone of our health systems… without them, most people wouldn’t get referrals or consultations” (064).

### Theme 3: Competing Responsibilities, Tensions, and Burnout

AHs described competing clinical, educational, and scholarly demands as a source not only of logistical strain but of identity strain, particularly when institutional structures limited their ability to enact roles aligned with their academic values. These pressures manifested through the expectation to volunteer for academic work before earning protected time, insufficient protected time for non-clinical engagement, pressure to produce scholarship without support, and structural isolation through night shifts.

#### Proving Yourself First — The Unpaid Labor of Legitimacy

Early-career AHs described a culture of uncompensated academic work. “If you want to do any extra teaching, if you want to do any extra publishing and extra research… any extra academic endeavors you pursue unfortunately are going to come out of your own private time” (035). Another reflected: “Earlier in my career, I would just volunteer for everything. And then I realized that realistically, there’s only so many hours in the day and that your time is so precious” (031). Legitimacy, in this framing, had to be earned through personal sacrifice rather than institutional investment.

#### Protected Time as Currency — Negotiating Sustainability

Protected time emerged as both a marker of institutional validation and a necessity for career sustainability. “Residency programs and academic environments want you to prove your work first and then they give you kind of bought down time later” (018). The week-on/week-off schedule was described as both a “blessing and a curse” – “it tends to be very inflexible on service weeks” (030) creating what one participant called a “feast or famine schedule” (029). Participants emphasized that off-service weeks were primarily for recovery: “When you work 7 days a week… by the time you’re done with your week, you’re pretty tired… You’re kind of recollecting yourself in your life” (065). Another added: “My job is both the clinician and both the academic and the hospitalist. So it’s a constant struggle to manage all this stuff” (005). Protected time was frequently described as key to “career sustainability and longevity” (030).

#### The Pressure to Produce

Scholarship was frequently expected but rarely supported with protected time. “I do feel sometimes there’s pressures to increase like research and scholarship activity. And when I try to incorporate that, I do feel some added pressure in term of time and work-life balance” (015). Many AHs experienced ambivalence about scholarship, questioning its relevance to their professional identity: “There was definite pressure to publish so that promotion can happen… until I realized I don’t really like doing scholarship… those benefits don’t apply to me and my goals” (005).

#### The Disruptive Weight of Nights

For participants whose role involved nights, nocturnal schedules disrupted academic engagement. “I don’t enjoy working nights… it’s a detriment to having exposure to residents and to medical students and to my non-clinical roles and all these things you need to engage in, those are all during the daytime” (031). Another elaborated: “It’s very hard to join project meetings or participate in education when you’re only up at night” (068). Another felt limited in their “ability to progress during those times that I was working nights” (029).

### Theme 4: Mentorship and Professional Growth

AHs described mentorship as central to professional growth and identity formation, yet access to meaningful mentorship remained limited and unevenly distributed. Consistent with identity theory, mentorship functioned as a core mechanism through which identity was negotiated, validated, and made visible, providing role modeling and belonging in the absence of clear institutional pathways. Participants described mentorship as both a personal need and a professional responsibility central to their academic identity.

#### The Mentorship Gap — A Young Field Still Growing

Hospital medicine’s relative youth has limited senior mentorship. “Hospital medicine is just a young field…is a very bottom-heavy field… we don’t have great long-standing deep roots of people” (030). Another described: “I’ve been an AH at three different institutions… in only one of those three places have I found like true mentorship and guidance in hospital medicine” (063). This participant explicitly linked mentorship to identity: “I feel like mentorship is going to be my next step in helping to establish my identity” (063).

#### The Impact of Strong Mentorship

When available, mentorship had substantial impact. “I’ve been very lucky… to have those mentors and just be really supported… not everyone has that great support” (023). Participants credited mentorship with shaping career trajectories: “I’ve had a really strong mentor… but if I did not have him, I would have zero shot” (065) in reference to their K award.

#### Mentorship as Professional Responsibility

AHs viewed mentoring learners as central to their academic identity. “As an AH you need to accept that you are a teacher, whether you’ve had formal training or not” (023). For many, mentoring provided meaning: “If you’re going to be in academics, it should be expected that you’re there to teach. But I think it’s also an expectation that you should be there as a mentor… they really do look up to you” (031).

#### Relational Fit and Representation in Mentorship

Effective mentorship required relational alignment, and participants emphasized the impact of mentors who shared identity or values. “Naturally we all gravitate towards people who are like us… if there is nobody who looks like you or sounds like you or shares your background, I think it requires more work to find the people that will mentor you” (030). One participant from an underrepresented background described deliberately seeking mentors with shared identity: “As a Black woman, my experiences are vastly different…so I’ve sought out mentors who look like me… that model the kind of physician I would like to be… that’s been responsible for a lot of my professional identity building” (054). Participants also emphasized that formal mentorship programs were often insufficient: “Mentorship is something that has to happen organically” (015).

## Discussion

Our analysis identified four interconnected themes shaping how AHs construct professional identity: endemic uncertainty about what constitutes “academic” work, tension between competing clinical and scholarly demands, critical mentorship gaps, and a persistent disconnect between how AHs view their contributions and how institutions recognize them. The presence of burnout despite early career stage suggests that identity fragmentation and institutional non-recognition of salient professional roles contribute meaningfully to emotional exhaustion and dissatisfaction.

### Interpretation and Meaning

Burke’s identity theory posits that a coherent sense of self develops through the roles individuals occupy and through social validation of behaviors aligned with those roles [19]. Our data suggest that in academic hospital medicine, institutional structures frequently prevent this alignment, producing “identity dissonance” the internal conflict that arises when individuals cannot enact roles consistent with their self-concept or when others fail to recognize them [19].

Many participants described “niche development”, anchoring themselves in secondary domains such as education, quality improvement, or leadership to create clarity and legitimacy. This parallels Varpio et al.’s framing of subjectification, where professional identities are made visible through unique, situated actions [23]. Notably, AHs’ identity construction, often through the educator role, occurred in spite of rather than because of institutional structures. Consistent with Burke’s identity theory, participants engaged in active identity work seeking verification, adjusting strategies, and renegotiating meanings in response to misalignment between institutional expectations and internal identity standards. While these efforts supported identity coherence, they were time-intensive and, participants emphasized, insufficient substitutes for consistent institutional recognition.

Protected time emerged as central to identity formation and sustainability. Participants described it not as a scheduling accommodation but as institutional validation of academic contribution. Without it, academic work becomes unpaid labor that contributes to burnout, a dynamic consistent with broader literature on clinician well-being [24]. Notably, burnout appeared even in early-career participants, challenging the assumption that it is primarily a later-career phenomenon.

### Contextualization with Existing Literature

Lang et al. identified mentorship of others as “currency for promotion” and emphasized its role in advancement [14]. Our findings extend this: mentorship is indeed essential, but remains scarce and unevenly distributed, particularly for underrepresented faculty. The mentorship gap reflects hospital medicine’s demographic youth as few senior mentors have spent entire careers as hospitalists. Mentorship is most effective when mentors are attuned to mentees’ evolving goals and identities, and when relational alignment, shared identity, lived experience, or values is present [25,26]. Mentorship tailored to underrepresented groups better equips faculty for advancement [27]. Our data also demonstrate that AHs’ mentorship identity extends beyond peers and junior faculty to learners, which participants described as central to their sense of purpose.

The expectation that AHs «prove themselves first» before receiving protected time has been described from the perspective of hospitalist group leaders, who frame early uncompensated academic labor as a prerequisite for later investment [28]. Our findings extend this by centering AH perspectives and showing the expectation operates across academic domains including teaching and is experienced as devaluing, contributing to identity dissonance and burnout.

Our findings on institutional non-recognition parallel Burke’s concept of identity verification the process through which behaviors aligned with an identity are socially confirmed [29]. This echoes prior work on medical educators, whose labor is often unseen and accorded lesser capital than clinical work [22]. We extend this literature to inpatient academic hospital medicine, where educational contributions are embedded in clinical service and rendered invisible by RVU-based valuation. When institutions fail to recognize teaching, mentorship, and non-billable work, they prevent verification of salient aspects of AH identity, producing frustration and identity fragmentation. Although learners consistently rate hospitalists as highly effective inpatient educators, this educator identity remains institutionally underappreciated [30].

### Limitations

This study was not designed to systematically examine how participant demographics influence PIF, and therefore participants did not consistently frame experiences through these lenses, except in the context of night work and mentorship with shared backgrounds. The sample was predominantly early-to mid-career, reflecting the bottom-heavy AH workforce, and it is plausible that PIF may manifest differently among senior faculty. Purposive snowball sampling through professional networks may have favored individuals more engaged with academic discourse. Finally, the study included only internal medicine AHs and does not capture the experiences of pediatric or family medicine hospitalists.

### Clinical and Practical Implications

*First*, institutions should develop clearer, field-wide definitions of AH work and communicate them explicitly at hiring and throughout tenure, while preserving flexibility for niche development. Definitions alone, however, are insufficient: participants described persistent structural tension in which institutions prioritize clinical service without embracing an academic vision for hospitalists. Without accompanying commitments to protected time, recognition, and advancement aligned with academic values, even well-articulated definitions fail to support PIF. Attention to flexible scheduling and promotion processes that account for those working nights is also warranted [31].

*Second*, protected time should shift from discretionary to non-negotiable, automatically provided at hiring rather than contingent on “proving yourself first.” The built-in week off in some AH schedules should be recognized as recovery time, not an expectation for additional academic work. Our findings suggest 20% protected time represents a meaningful investment, though most institutions in this study did not offer it.

*Third*, institutions should establish mentorship infrastructure that supports organic relationship formation while actively addressing gaps for underrepresented faculty, including dedicated mentorship roles with protected time, peer mentorship structures, and visible mentors from diverse backgrounds.

*Finally*, leadership should formally recognize AH contributions in teaching, mentorship, and education through tangible mechanisms: educational metrics in performance evaluation alongside RVU-based measures, institutional resources for educational initiatives, and compensation structures that account for the full scope of AH work.

Taken together, our findings suggest AHs should be understood as core academic clinicians whose educational, clinical, and relational work is central to institutional missions. When “academic” work is defined by RVU-driven metrics privileging scholarship and procedural productivity, teaching, mentorship, and clinical leadership are marginalized. Without recalibration, AHs are left to individually negotiate identity and legitimacy within systems that structurally constrain their contributions.

Future research should evaluate the effectiveness of institutional interventions standardized role definitions, guaranteed protected time, and relationally aligned mentorship programs on engagement, satisfaction, and retention. The paradoxical relationship between AHs’ younger age and higher burnout also warrants further exploration.

## Conclusion

AHs occupy a critical yet underdefined space in medical education. Their professional identities are shaped by persistent ambiguity, competing demands, and inconsistent institutional recognition, requiring ongoing individual effort to sustain coherence and legitimacy. However, identity cannot be left to individual work alone. Aligning institutional structures with the realities of AH contributions through clearer role definitions, protected time, mentorship infrastructure, and recognition of educational labor is essential to support their development, well-being, and retention.

## Additional File

The additional file for this article can be found as follows:

10.5334/pme.2758.s1 Supplementary material 1. Interview Guide.

## AI Declaration

The authors used Co-Pilot and Claude Opus 4.7 while finalizing the manuscript (accessed in March–May, 2026). The tools were used to provide language and grammar suggestions to refine sections of the text. All conceptualization, interpretation, and argumentation were developed by the authors. The authors critically reviewed, revised, and verified all AI-assisted content, including checking factual claims against source materials to ensure accuracy, originality, and coherence with journal guidelines. The authors take full responsibility for the final content and interpretations.
