# Supplementary material for: Essential Yet Invisible: Professional Identity Formation Among Academic Internal Medicine Hospitalists
Source: Perspect Med Educ. 2026 Jun 19;15(1):534–45. doi: 10.5334/pme.2758 (PMC13281736; doi:10.5334/pme.2758)
Supplement: Supplementary material 1. — Interview Guide. [file pme-15-1-2758-s1.pdf]

Supplementary Material 1

Interview Guide

Introduction:

I am XYZ. Thank you for participating in this research interview. We are interested in gaining insights into your professional experiences as an academic hospitalist (AH), specifically focusing on your professional identity formation (PIF) and your perceived roles in inpatient education. Medical PIF is a journey of personal development and social construction. It is the dynamic process of enculturation into the actions, interactions, values, and norms of the medical profession. Your input is invaluable in understanding this critical aspect of healthcare and medical education.

Probe 1:*("Being" an AH)*

Can you describe your journey and motivations in becoming an academic hospitalist (AH)? Please share the story of your academic journey.

Sub Probes:

1. What does "being" an academic hospitalist mean to you? How do you perceive your professional identity as an academic hospitalist?

2. What factors have influenced the development of your identity as an AH?

3. Have there been any pivotal experiences or mentors who have shaped your professional identity?

4. As you likely are aware there are debates about the roles an AH should have as a faculty member at an institution. What do you think the role of an AH should be compared to a hospitalist who is not required to engage in teaching activities?

5. As an AH what pillars of the educational mission at you institution do you feel most drawn to (if any) and why (clinical service, teaching and education, scholarship and research etc)?

6. If you have an “underrepresented in medicine” (URiM) identity (URiM includes minoritized secondary to race, ethnicity, gender, sexual preferences, socioeconomic factors, first-generation college status), do you feel that this plays a role in your career development as an AH? If so, please tell us how?

7. As an AH have you experienced any professional bullying? Can you describe the incident (if comfortable sharing)?

8. Do you work nights as part of your role as an AH?

1. If yes- how does your role working nights contribute to your identity as an AH?

Probe 2: *(Perceived Roles in General)*

9. Other than as a clinician, what other responsibilities do you have on a daily basis? (Examples include leadership, administrative, educational, research). How were you drawn into these roles? And do feel these roles impact the way you see yourself as an AH who is core to an academic institution?

Sub Probes:

10. Of these roles and responsibilities which do you dedicate the most time to (including clinical)? Would you like to be involved in other roles? If so what roles?

11. What specific interests do you have outside of medicine and how do these impact your PIF as an AH?

12. How do relationships with peers and friends/family (outside of your institution) impact your work identity as an AH?

Probe 3: *Perceived Roles in Inpatient Education*

What roles and responsibilities do you believe AH should have in the education of residents and students on the inpatient service? Do you a percent of time/effort dedicated to teaching? If so, what is the format of the teaching activity?

Sub Probes:

13. What experiences do you have in the past in teaching UME or GME? Prior to this position, had you worked in an academic institution with direct educational responsibilities of residents or students? How have these experiences shaped your identity as an AH?

14. What specific educational interests do you have? And have you had a chance to be involved in educational scholarship? If so, please give us an example.

15. How do you feel about balancing your clinical duties with your teaching, research or other responsibilities? Do you feel a tension between these responsibilities? And what impact has this tension had on your identity as an AH?

Probe 4: (Interactions and Collaborations)

How have peer interactions shaped the way you view yourself in the academic enterprise at your institution?

Sub Probes:

16. AH often collaborate and interact with other members of the healthcare team, such as attending physicians, nurses, and allied health professionals. How have these instances shape the way you view yourself and your role as an AH?

Probe 5: (Adaptation and Future Directions)

With the evolving landscape of healthcare and medical education, how do you see the role of academic hospitalists in learner education changing in the future?

Sub Probe:

17. What strategies or innovations do you think can enhance the effectiveness of academic hospitalists and help them build an identity of being an AH?

18. Are there any resources or support systems that you believe would improve your ability to fulfill your roles in learner education?

Closing:

Thank you for sharing your valuable insights. Your perspectives will contribute to our understanding of the challenges and opportunities in the field of academic hospital medicine. If you have any additional comments or suggestions, please feel free to share them.
